# Early adoption of innovation in HPV prevention strategies: closing the gap in cervical cancer

**DOI:** 10.3332/ecancer.2024.1762

**Published:** 2024-09-11

**Authors:** Ishika Mahajan, Amogh Kadam, Lucy McCann, Aruni Ghose, Katie Wakeham, Navjot Singh Dhillon, Susannah Stanway, Stergios Boussios, Soirindhri Banerjee, Ashwini Priyadarshini, Bhawna Sirohi, Julie S Torode, Swarupa Mitra

**Affiliations:** 1 Department of Oncology, Lincoln County Hospital, United Lincolnshire Hospitals NHS Trust, Lincoln, Lincolnshire, UK; 2 Government Cuddalore Medical College and Hospital, Chidambaram, India; 3 Department of Oncology, Barts Cancer Centre, St. Bartholomew’s Hospital, Barts Health NHS Trust, London, UK; 4 Centre for Primary Care, Wolfson Institute of Population Health, Queen Mary University, London, UK; 5 Department of Medical Oncology, Medway NHS Foundation Trust, Gillingham, Kent, UK; 6 Centre for Cancer Biomarkers and Biotherapeutics, Barts Cancer Institute, Queen Mary University of London, London, UK; 7 United Kingdom and Ireland Global Cancer Network; 8 Prevention, Screening and Early Detection Network, European Cancer Organisation, Brussels, Belgium; 9 Radiotherapy UK; 10 Department of General Surgery, Pilgrim Hospital, United Lincolnshire Hospitals NHS Trust, Boston, Lincolnshire, UK; 11 Department of Medical Oncology, The Royal Marsden NHS Foundation Trust, London, UK; 12 Faculty of Life Sciences and Medicine, School of Cancer and Pharmaceutical Sciences, King’s College London, London, UK; 13 Kent and Medway Medical School, University of Kent, Canterbury, UK; 14 Faculty of Medicine, Health and Social Care, Canterbury Christ Church University, Canterbury, UK; 15 AELIA Organisation, Thessaloniki, Greece; 16 Department of Radiation Oncology, IPGMER and SSKM Hospital, Kolkata, India; 17 Department of Preventive and Social Medicine, VMMC and Safdarjung Hospital, New Delhi, India; 18 Department of Medical Oncology, BALCO Medical Centre, Vedanta Medical Research Foundation, Chattisgarh, India; 19 Institute of Cancer Policy, Centre for Cancer, Society & Public Health, King’s College London, London, UK; 20 Department of Radiation Oncology, Fortis Medical Research Institute, Gurgaon, India; † All authors contributed equally; * Joint Senior Authors

**Keywords:** cervical cancer, screening, HPV, Pap, primary prevention, secondary prevention, vaccine, population health, elimination

## Abstract

Cervical cancer (CC) is one of the highest prevailing causes of female cancer-related mortality globally. A significant discrepancy in incidence has been noted between high and low-middle-income countries. The origins of CC have been accredited to the human papillomavirus (HPV) with serotypes 16 and 18 being the most prevalent. HPV vaccines, with 90%–97% efficacy, have proven safe and currently function as the primary prevention method. In addition, secondary prevention by timely screening can potentially increase the 5-year survival rate by >90%. High-precision HPV DNA testing has proven to be both highly sensitive and specific for early detection and is advocated by the WHO. Lack of public awareness, poor screening infrastructure and access to vaccines, socio-cultural concerns, along with economic, workforce-associated barriers and the presence of marginalised communities unable to access services have contributed to a continued high incidence. This article comprehensively analyses the efficacy, coverage, benefits and cost-effectiveness of CC vaccines and screening strategies including the transition from cytological screening to HPV self-sampling, while simultaneously exploring the real-world disparities in their feasibility. Furthermore, it calls for the implementation of population-based approaches that address the obstacles faced in approaching the WHO 2030 targets for CC elimination.

## Introduction

**Figure 1.**
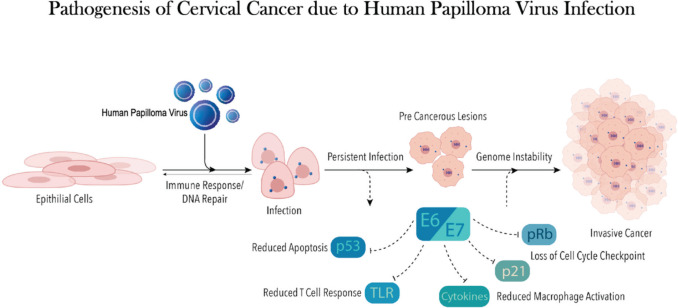
This figure shows onco-pathogenesis of CC due to HPV.

Cervical cancer (CC) is the fourth highest cause of cancer-related mortality among women worldwide. In 2020, 604,100 new CC cases were reported with over 341,000 fatalities [1]. The human papilloma virus (HPV), a DNA virus, is the predominant causal agent of CC [2] (Figure 1). Twelve oncogenic strains of HPV have been identified as group 1 carcinogens or high risk by the International Agency for Research on Cancer [3], with serotypes 16 and 18 accounting for about 70% of CC cases [4].

Significant disparities between economically developing and developed countries exist regarding incidence (18.8 versus 11.3 per 100,000) and mortality (12.4 versus 5.2 per 100,000). This variance is directly driven by the disproportion in the availability of prevention and screening technologies and resources [5]. Ninety percent of deaths attributable to CC occur in low- and middle-income countries (LMICs), especially in sub-Saharan Africa, Melanesia, South-East Asia and South America [6]. Malawi in sub-Saharan Africa has the world’s highest CC incidence and mortality [7].

CC prevention requires a dual-pronged approach via screening and vaccination. Vaccination or primary prevention of CC started in 2007, but by 2020 it had been introduced as a prevention strategy in only 57% of countries worldwide [8, 9]. The full effect of vaccination can be seen only in the years following implementation as it prevents the natural history of the disease.

Screening achieves short-term goals through early detection of CC or premalignant lesions [10]. In the long term, sustained high coverage of vaccination, creates a population that is protected from high-risk diseases. There is thus a decreased screening requirement [11].

Despite the possibility of being able to achieve near complete prevention of disease, efforts to lower the incidence to the global elimination goals are forestalled, broadly due to a lack of public awareness or poor access to the vaccine, poor screening program infrastructure and insubstantial utilisation of strategies in LMICs secondary to cost and human resource factors [6, 12].

In a groundbreaking effort to eliminate CC entirely, the WHO launched a global strategy in November 2020. This ambitious plan, known as the ‘90-70-90 strategy’, aims to vaccinate 90% of girls against HPV by age 15, ensure 70% of women have access to accurate and reliable screening tests for CC by age 35 and again by 45 and guarantee 90% of women diagnosed with CC receive proper treatment, including both pre-cancerous and invasive stages, by the year 2030 [13].

**Figure 2.**
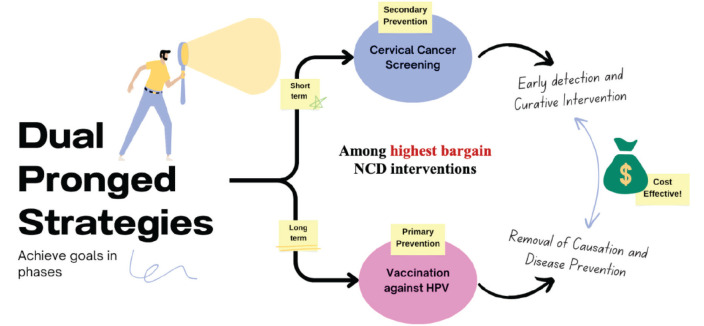
This figure is a conceptual diagram summarising the two main strategies for CC prevention.

Our review article comprehensively impresses on population-based strategies for primary and secondary prevention of CC (Figure 2). It also explores the hurdles in the path towards achieving the WHO 2030 targets, paving the way to elimination. We also aim to elaborate on the success of vaccination and screening programs in some countries, hoping to extrapolate these to the high-burden, low-reach zones.

## HPV vaccination: the primary prevention

Primary prevention strategies against CC focus on prophylactic vaccination as the most effective form of long-term primary prevention of CC [14].

Three HPV vaccines are used most widely: Cervarix (a bivalent vaccine targeting HPV 16 and 18), Gardasil (a quadrivalent targeting 6/11/16/18) and Gardasil 9 (nonavalent targeting 6/11/16/18/31/33/45/52/58). These prevent about 70% to 90% of CC cases depending on the type used [15–17]. Recent developments have led to multiple other vaccines completing clinical trials in China and India (Table 1).

Since first licensure in 2006, HPV vaccination has been recommended for global use in females between 11 and 12 years by the US Centers for Disease Control and Prevention (CDC)’s Advisory Committee on Immunisation Practices [8, 9]. Over time, emerging evidence has led to adaptation of these global vaccination recommendations to improve cost-effectiveness and coverage. Recent evidence from Scandinavian countries suggests optimal effectiveness around adolescence or younger [18]. Beyond the age of 26, the efficacy and cost-effectiveness are poor, as people have often already potentially been exposed to HPV [19].

HPV is implicated in several non-CCs in both women and men, specifically anal, oropharyngeal, penile, vaginal and vulvar cancer. Even with the introduction of a national vaccine program for HPV for women and girls in most developed countries, the male vaccine program is yet to be established as a stronghold for prevention [20]. However, in 2016 WHO formulated public health recommendations which were extended to include vaccination rollout for boys and catch-up schemes for adults due to its cross-cover for non-cervical pathologies. This was based on high coverage in girls, i.e., ≥80% to be the most cost-effective means to achieve herd effect [21]. As per 2022 data, Canada, Denmark, Norway and Chile had achieved ≥ 80% coverage of first-dose vaccination in boys, with the USA, Argentina, Australia, Italy, New Zealand and the UK following behind with >50% coverage [22].

### Efficacy

The efficacy of two- and three-dose HPV vaccination regimens have been rigorously assessed in large cohorts, both pre- and post-licensure. Initially, the WHO recommended a 3-dose schedule. In 2014, this shifted to a two-dose series spaced 6–12 months apart. More recently, a review of the evidence led to the Strategic Advisory Group of Experts on Immunization supporting both single and two-dose HPV vaccination schedules due to their comparable individual effectiveness and ease in programme implementation [23].

In 2018, the WHO commissioned an independent systematic review to assess the potential protection of the vaccine and the benefit it has on public health [24]. Across 26 trials (73,428 participants), they concluded with high-certainty evidence that HPV vaccines are protective against cervical precancers in adolescent females aged 15–26. As per protocol analysis, all licensed vaccines have proven similar successful efficacy [25]. However, Gardasil 9 has the potential to prevent 90% of CCs due to the additional strains it covers [26]. In addition to protecting the individual, there is also strong evidence that HPV vaccination offers herd immunity when sufficient population coverage is reached, further enhancing its efficacy [11].

The Kenya single-dose HPV-vaccine efficacy trial found that a single dose of Gardasil 9 produced an efficacy of over 97%, which was similar to that found in their trial assessing the triple dose regimen [27]. Early data from the Dose Reduction Immunobridging and Safety Study trial supports these findings, demonstrating that antibody avidity is the same in groups treated with single-, two- and three-dose regimens [28].

### Safety

HPV vaccines have been consistently proven safe for over 15 years in rigorous independent analyses of large cohorts. This has included regular assessment by the Global Advisory Committee on Vaccine Safety from 2007 to 2015, which has repeatedly concluded no evidence of association with adverse effects [29]. In an independent analysis, deaths within the vaccine were deemed to be unrelated to the administration of the vaccine [24].

### Coverage and effectiveness

As with many public health strategies, efficacy and safety in a clinical trial setting do not equate to effectiveness in real-life settings. Despite its licensure over 15 years ago, population coverage remains suboptimal. Current public HPV programmes cover less than a third of those eligible for the vaccine [29, 30]. A combination of barriers has led to poor uptake of vaccines, including problems with demand, supply and affordability which vary substantially depending on the setting.

Where vaccine programmes have been implemented effectively, the benefits are already seen. The UK has one of the most effective national vaccination programmes globally. In the 13 years since its implementation in 2008, it has substantially reduced CC incidence to near elimination [31]. However, this is not the case globally. While the majority of high-income countries (HICs) have >75% coverage, there remain some which have lagged. Japan is an HIC which has seen dramatic changes in vaccine uptake. The changes in recommendations from the Ministry of Health in 2013 led to a decline in coverage to a low of 0.3% in 2016 [32]. Since then, rectifications in recommendations in 2021 mean that the rates have improved [33]. We are yet to see any cancer spikes due to the lag time between HPV infection and CC presentation. However, this waiver in vaccine implementation is estimated to have resulted in around 5,000 preventable deaths [32].

**Figure 3.**
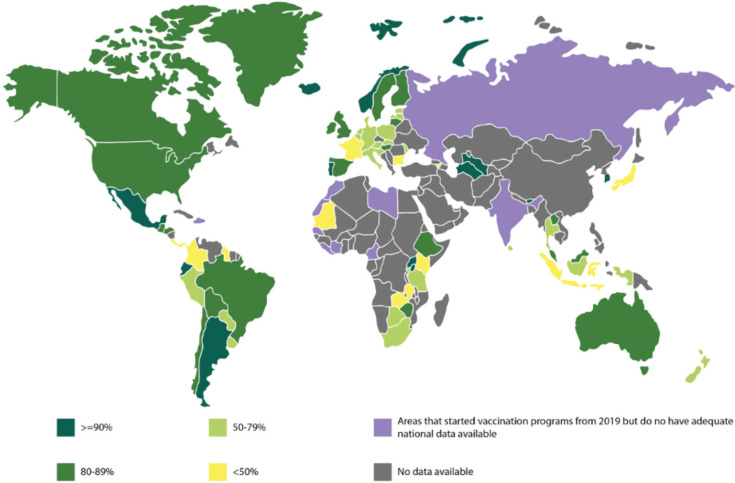
The world map above shows the available data for vaccination cover of the first dose of the HPV vaccine in girls till the year 2022 [22, 30] (The areas shaded in purple depicts the areas which have started vaccination programs from 2019, but do not have adequate national data available).

Globally, there is vast inter- and intra-country variation, with the most significant disparity being between LMICs and HICs (Figure 3). Vaccination coverage tends to correlate with socioeconomic deprivation both between and within countries [34, 35]. In many LMICs, initial dose coverage of >70% was seen; however, this dropped with subsequent doses, with the third dose coverage being only around 30%. The ill-sustained impact is likely due to funding momentum failing and a dearth of sustainable programmes [36]. Furthermore, there is a large vaccination gap between urban and rural areas. Bolivia and Honduras are both exceptions which have achieved almost two-thirds coverage, sustainably maintained [34, 37]. All ten countries with the highest age-standardised rates of CC mortality are within West Africa, where national vaccination implementation has been slow [38]. This stems from a poor vaccine acceptance rate including poor information, safety, fertility and promiscuity concerns. In contrast, in Southeast Asia, vaccine uptake increased significantly once it was offered for free [39]. Altogether, the barriers to successful vaccination programs in LMICs are complex and vary depending on multiple factors including cultural, financial and environmental [34, 35].

The COVID-19 pandemic significantly disrupted routine vaccination programs worldwide. Studies from countries such as Brazil and the United Kingdom documented substantial declines in HPV vaccine doses administered during lockdowns and social distancing measures [40, 41]. A modelling study in the US predicted a 75% reduction in HPV vaccination during the first wave of COVID-19 [42].

Disruptions stemmed from several factors. School closures, a common strategy to curb COVID-19 spread, hindered school-based vaccination programs, a crucial delivery channel for HPV vaccines [42]. Healthcare system resource constraints further hampered routine vaccinations, with staff and facilities diverted to COVID-19 response efforts [43]. Additionally, vaccine hesitancy may have increased due to pandemic anxieties and misinformation [42].

Despite the challenges, some countries like Israel maintained or even increased HPV vaccination rates during the pandemic [44]. This suggests the importance of robust vaccination programs and effective communication strategies to mitigate disruption.

Even after restrictions have been lifted, the pandemic has continued to have residual disruption to vaccine supply and demand [45]. Recovery efforts are underway. The WHO emphasizes the need for catch-up programs to address vaccination gaps and restore pre-pandemic coverage levels [43]. Additionally, promoting HPV vaccination alongside COVID-19 vaccination campaigns can improve efficiency and reach [46].

### Cost effectiveness

The cost-effectiveness of HPV vaccinations has been proven consistently. In a multi-country meta-analysis Rosettie *et al* [47] assessed incremental cost-effectiveness ratios (ICERs) for HPV vaccination in 195 countries and found a mean of US$ 4,217 per DALY averted (95% uncertainty interval: US$773–13,448) globally. This was significantly lower for certain regions, particularly in LMICs. ICER was reduced with values of US$706 per DALY averted (95% UI: $130–2,245) in Southern Asia and Sub-Saharan Africa [47]. This means that although individual mortality and morbidity benefits may be modest, there is substantial financial benefit to the implementation of HPV vaccinations at a global level.

Notably, the level of cost-effectiveness can vary depending on various factors including vaccine delivery method, number of vaccines given and target population, with young women and men having sex with men being the most effective cohort. Similarly, vaccinating those at older ages provides less cost-effectiveness due to the limited benefit [48].

Using the UK as a case study, an analysis was provided by Datta *et al* [49] Vaccinating girls provides significant health benefits at a reasonable cost. Threshold prices of £56–£108 per dose imply the healthcare system would see this as worthwhile. With a lower discount rate (1.5%), i.e., when future health benefits of preventing penile and throat cancers are valued more highly, vaccinating both sexes becomes cost-effective. At a higher discount rate (3.5%), vaccinating both sexes is less cost-effective than girls alone (£25–£53 threshold price). This is mainly due to herd immunity: vaccinating girls significantly reduces HPV transmission, indirectly protecting boys [49].

As discussed previously, various alterations of vaccination strategies have been made in recent years to improve ICER, such as the delivery of a single vaccination and combining vaccine delivery with that of other vaccinations [50].

Another consideration of cost-effectiveness is the cost per unit of each dose. Unit dosing is dependent on several factors, further complicating cost-effectiveness decisions within countries. For instance, the nonavalent vaccine Gardasil 9, is priced higher for full doses compared to the bi- and quadrivalent cervical vaccines. This means that in some countries, it is not cost-effective when the other vaccination options are, despite its coverage over more strains [51].

Similarly, prices of the same vaccine vary between different countries. While those within the GAVI Alliance are offered a sustainable supply of low-cost vaccines, the same vaccines can be over 20 times more expensive in developed countries [34]. Thus, countries such as Vietnam rely on qualifying for GAVI-priced vaccines to have sufficient cost-effectiveness to justify funding cervical vaccination programmes [52].

Middle-income countries that do not qualify for GAVI subsidies are most at risk of not being able to afford comprehensive vaccination programmes, often referred to as the ‘missing middle’. Despite the limited support, by 2020 around half of these countries had managed to implement a vaccination programme, with many more developing pilots [51]. In 2022, India achieved market authorisation of the novel vaccine Cervavac, which covers the same four strains as Gardasil. This offers a more affordable vaccine to India’s population; costing approximately 200–400 rupees (or 3.5 EUR), compared to the alternatives which are around 10 times the price [53, 54]. Such opportunities must be welcomed and encouraged to maximise protection.

Despite its proven safe, efficacious existence for a significant time, the coverage and cost-effectiveness of the HPV vaccines remain substantially sub-optimal.

### People living with HIV (PLHIV)

HPV vaccination is crucial in PLHIV due to their increased susceptibility to HPV-related cancers. The WHO, the CDC and the British HIV Association recommend HPV vaccination in this population [55–57].

Studies have shown that the HPV vaccine is immunogenic in PLHIV [58, 59]. However, the magnitude of the immune response may be lower compared to the general population, particularly in individuals with advanced HIV disease or low CD4 counts [59, 60].

Available evidence suggests that the HPV vaccine can still be protective against HPV infection and related pre-cancerous lesions in PLHIV [60, 61]. However, long-term efficacy data and data on the prevention of invasive cancers specifically in this population are still limited [60, 61]. Ongoing research is crucial to fully understand its long-term impact and the potential need for different vaccination strategies in this population.

PLHIV are recommended to receive HPV vaccination according to the standard recommendations for the general population (typically 2 or 3 doses depending on age at initiation). However, some guidelines, like the CDC’s, recommend initiating vaccination as early as 9 years of age for individuals with HIV, considering potential immune system compromise [56]. The Canadian Paediatric Society suggests a 3-dose regimen for immunocompromised individuals to ensure optimal immune response [62]. Despite the guidelines, achieving optimal HPV vaccination coverage among HIV-positive individuals remains a challenge.

## CC screening: the secondary prevention

Secondary prevention or screening programs aim to reduce morbidity and mortality associated with CC by increased detection of precursor lesions and early identification of invasive carcinoma [63]. If recognised early, the 5-year survival rate of women diagnosed with invasive CC is 92% as compared to 58% and 18% for locally advanced and metastatic stages, respectively [1].

## Screening tools: transition from cytology to HPV testing

### The Pap smear

Since its introduction in the 1940s, papanicolaou smear has been the most widely used tool for the detection of precancerous cervical lesions. It led to a greater than 70% decrease in the mortality associated with CC in the US [64]. However, a nationwide study in the UK found its sensitivity to be 55.4% and specificity to be 96.8% [65]. A meta-analysis further compared 11 separate studies with a cumulative of 39,050 participants to find that sensitivity and specificity estimates of cytology ranged from 0.43 to 0.94 and 0.78 to 0.98, respectively. The pooled sensitivity and specificity were 0.70 and 0.95, respectively. While specificity remained consistent over several studies, the sensitivity was widely variable [66]. To balance this inconsistency in sensitivity, a lifetime of repeat testing is warranted. In low-resource settings where regular testing is difficult due to limited access and knowledge about CC screening, this poses a significant hurdle. Another caveat is their implementation. Pap smears are labour intensive and require repeat testing, trained personnel to conduct and interpret the test, equipment and other resources. All of these are a lacuna in many regions of the world [67]. Adoption of a cytology (Pap smear)-based screening program in a few LMICs has shown little benefit, with an unchanged mortality rate after 5 years. This was likely due to the above-mentioned constraints, low-quality smears and the inability to allocate adequate capital to ensure high-quality standards [68].

### Visual inspection

To overcome the aforementioned gap and to adopt a screen-and-treat approach, visual inspection with acetic acid (VIA) became the preferred CC screening modality in LMICs. The sensitivity and specificity estimates after comparing 11 studies were found to be 0.17–1.00 and 0.08–0.95, respectively. The pooled sensitivity and specificity were 0.77 and 0.82, respectively [66]. Visual inspection techniques can be performed relatively more efficiently than other modalities in low-resource settings as they require a simpler infrastructure, are not specialist-intensive and are significantly cheaper. Other advantages for high-burden zones are that VIA only takes a few minutes to perform, and the results can be immediately interpreted, negating the need for time-consuming laboratory processing [69]. It was also found that visual inspection methods had higher sensitivity than cytology testing, detecting more high-grade CIN in several studies conducted in India [70]. However, this ability to detect a greater number of positive cases is a double-edged sword. It was found that when compared to cytology or HPV testing, VIA led to a much higher number of false positives and subsequent overtreatment [66]. While VIA appears to be a good alternative to the Pap smear in LMICs, it comes with significant challenges of scaling up. A variance of sensitivity is also seen in older women with other endocervical lesions. Moreover, factors such as age, parity and underlying cervical disease also affect the positive predictive value [70]. There is also a significant difference in accuracy when visual inspection is performed by physicians (30.2% positivity) versus nurses (36.3% positivity), highlighting the significance of the subjective nature of the evaluation [71]. Vedantham *et al* [72] uncovered a remarkable difference in positivity rates (4% to 31%) when similarly qualified gynaecologists conducted testing and the greatest variation was seen between the two doctors that had received the most training. They also found that the positivity rates increased for disease-negative women with cervical inflammation (6.1%–15.5% (*p*-value < 0.001) [72].

### HPV testing

The shortcomings of cytology and visual inspection techniques along with the realisation that oncogenic strains of HPV are responsible for over 95% of CC led to the introduction of HPV DNA testing in CC screening programs [73] (Table 2).

Initially, HPV assays were integrated into clinical practice to triage minor cytological abnormalities, then along with cytology as co-testing and now most recently as a stand-alone primary screening tool [69]. The latest WHO guidelines recommend HPV DNA detection as the modality of choice for primary CC screening in women of the general population and also those that live with HIV, over cytology and VIA. It advocates the screening to begin by the age of 30 and until age 50 with screening intervals of 5 or 10 years [74]. An HPV DNA test looks for the presence of viral DNA in the cervical cells [75]. HPV testing is comparable in accuracy to the other commonly employed modalities for the detection of cervical precancerous lesions among women. It detects high-grade precancerous lesions (CIN3) earlier than cytology, thereby increasing the likelihood of treatment before progression to invasive cancer. A follow-up of four randomised control trials found that HPV testing offers 60%–70% more protection against invasive cancer than cytology [76]. Additionally, a single round of HPV DNA testing has been shown to increase the number of cases detected and lead to a reduction in the progression to advanced cancer and mortality [77].

The sensitivity and specificity of singular HPV DNA testing were found to be 0.64–1.00 and 0.56–0.97, respectively. The pooled sensitivity of 15 studies was 0.94 and specificity was 0.88 [64]. The ATHENA Study conducted in the US found that HPV testing detected 64.2% more CIN3 when compared to cytology and 22.5% more CIN3 than a co-testing strategy. This stark difference was seen when baseline testing was done and also on an annual follow-up done for 3 years subsequently. It was also noted that HPV Testing had the highest 3-year adjusted sensitivity when compared to cytology (47.8%); and co-testing (61.7%) for CIN 3. Moreover, a negative HPV test had a much lower risk of CIN3 as compared to a negative cytology report. Out of all the women with a positive CIN3 over 3 years, 47.3% had an initial negative cytology report while only 9.8% had an initial negative high-risk HPV test [78].

## Co-testing versus stand-alone testing

Co-testing refers to testing for HPV DNA and cytology from a single sample collected by a clinician. It takes longer and is more expensive when compared to a single HPV test alone. HPV testing had a sensitivity of 90% and 95% for detecting underlying CIN2+ and CIN3+, respectively. Adding cytological testing to it leads to an increase in average sensitivity of only 5% for CIN2+ and 2% for CIN3+ along with a marked decrease in the specificity 0.95 and 0.93, respectively [79]. The added sensitivity benefitted very few women, as little as five women per million per year [80]. Another study found that in comparison, stand-alone testing was superior to co-testing using both Pap smear and liquid-based cytology. Co-testing with Pap also had higher false positive results and more referrals to colposcopy [81]. A study that compared the long-term emergence of pre-cancer and cancer in negative tests found that primary HPV tests done every 3 years provided similar, if not more, reassurance than co-testing every 5 years. The risk for HPV testing and co-testing was as follows, CIN3: 0.069% versus 0.19%, (*p* < 0.0001) and invasive cancer: 0.011% versus 0.020%, (*p* < 0.0001) [82]. There is an insignificant difference in the cumulative risk of invasive carcinoma for a double negative result (HPV and cytology) as compared to a single HPV negative result in the long run [79]. Additionally, an HPV-negative/cytology-positive result only led to a minor increase in the detection of precancerous lesions (3.5%) and cancer (5.9%) [80].

The small increase in sensitivity afforded by co-testing is inadequate to justify the difficulties posed by it, thus stand-alone HPV testing should be considered as the superior screening tool over co-testing.

## DNA versus mRNA testing

WHO recommends countries that are starting screening programs begin with HPV testing and encourages others to transition to the same as soon as feasible. However, HPV mRNA testing can be a suitable alternative. The increased expression of the mRNA coding for E6/E7 proteins, which are essential for CC pathogenesis, shows a higher risk for cancer. These tests primarily detect the presence of E6/E7 mRNA of oncogenic HPV types and thus could be a more specific predictor for cancer risk [83]. In comparison to DNA testing, mRNA testing was discovered to be similarly sensitive and more specific with a relative sensitivity and specificity of 0.98 and 1·03 for CIN2+. A 4–7-year relative sensitivity analysis showed similar values in the range of 0.91–1.05 when compared to DNA testing. The commonly used APTIMA test had a lower sensitivity for self-collected samples and hence use could be limited to clinical settings [84].

The consideration of mRNA in primary CC screening is due to its increased specificity compared to DNA testing. A few separate studies that evaluated the role of mRNA as the primary screening tool found that it produces results similar to HPV DNA testing programs [85, 86]. However, a reduction in colposcopy referrals that is expected of a modality with higher specificity was not observed [86]. That combined with the finding that solitary mRNA screening would miss 3% of patients with CIN 3 when compared to DNA testing has led to some reluctance to shift to mRNA testing [87]. An interesting application was noted by a group that tried mRNA triage on DNA-positive cases. Both tests were performed on the same sample. It was found this co-testing strategy led to a 54.5% reduction in colposcopy referrals and was more cost-effective when compared to stand-alone DNA testing [88]. The most conclusive evidence for the debate between mRNA versus DNA comes from the WHO recommendations which firmly advocate for the HPV DNA test as the first-line primary screening tool. It suggests that mRNA may be used as an alternative when the samples are collected by the clinician and there exists the capacity to repeat testing at 5-year intervals [74].

## Self sampling

HPV self-sampling (HPV SS) is a process by which a woman can use a testing kit (options available to programmes include lavage, brush, swab, or vaginal patch) to collect a cervicovaginal sample that is sent for laboratory analysis. The test cannot diagnose CC but will identify if a woman is infected with a high-risk HPV by detecting viral DNA [89]. Rolling out self-sampling kits in bulk as part of the CC screening program led to an increase of 10% in test coverage in just a year in Sweden. It was well accepted by the target population due to its ease of use and is recommended due to the lower costs associated with it [90].

Non-responders are screening-eligible women who do not participate in routine CC screening programs. Non-responders are significant because 50% of all positive cases from the UK, USA and Netherlands are seen in women with no history of screening. HPV SS can be a possible solution to fill this gap [91]. Many women are reluctant to participate in clinical conducted programs because there is a fear of pain, embarrassment and invasion of privacy, particularly related to the pelvic examination using a speculum. Often, religious/cultural sentiments and a lack of access to healthcare centres prevent interested women from attaining necessary screening interventions. HPV SS is a unique solution to this multifaceted problem because the test can be done easily, in private and without any pain [92].

A 2014 meta-analysis that pooled data from 36 studies revealed that HPV SS could detect 76% of CIN2 and CIN3. The pooled absolute specificity for CIN2+ was 86% and for CIN3 was 87% [93]. The pooled sensitivity and specificity of self-collected samples were slightly lower than that of clinician-collected samples (12% reduction in sensitivity for CIN2+) [94]. This gap can further be bridged by the usage of polymerase chain reaction (PCR)-based HPV testing which has similar sensitivities across both scenarios [93].

The MARCH randomised control trial assessed the value of HPV SS and compared it to cytology in Mexico. They found HPV SS to be 3.4 times more sensitive and it detected 4.2 times more invasive cancer than cytology. One drawback was the lower positive predictive value of the test, but it makes up for it with higher sensitivity. In low-resource settings, cytology-based programs are not as successful due to restricted infrastructure. Women in these regions typically get tested only a handful of times in their lifetimes and hence benefit from a more sensitive test [95].

Due to its comparable clinical accuracy, lower cost and better acceptability, HPV SS can potentially become an important add-on strategy for women who do not participate in routine screening.

### A population-based cervical screening program

Population-based screening programs require that a target group be identified and invited to participate. In opportunistic screening, the healthcare provider or patients must take the initiative to do screening as per the guidelines (Table 3) [96]. The WHO advocates for the adoption of large-scale, national population-based screen-and-treat programs. The present guideline demands for screening at least 70% of women by the age of 35 and then again by 45 (with 90% follow up when cervical disease is identified), working toward a 2030 goal of elimination of CC [6, 97, 98].

The WHO CC Elimination Modelling Consortium was developed in 2020 and includes the 90-70-90 triple intervention coverage targets, i.e., scaling up of HPV vaccination, twice-lifetime cervical screening, treatment of CIN and invasive cancer to 90%, 70% and 90%, respectively [6, 97, 98]. This model was used to predict CC-related mortality in 78 LMICs. Vaccination alone would reduce mortality by 0.1%, 61.7% and 89.5% by 2030, 2070 and 2120, respectively. Integrating twice lifetime screening and treatment would increase those figures to 34.2%, 92.3% and 98.6%, respectively. Moreover, by 2030, half and one-third of CC related deaths could be avoided in sub-Saharan Africa and South Asia respectively, in sync with the UN sustainable development goals [97]. In another analysis of the model, girls-only vaccination (age 9 at catch up to age 14) yielded elimination in 60% LMICs with threshold incidence ≤4/100,000, 99% LMICs with incidence ≤10/100,000 and 87% LMICs with ≥85% reduction of threshold incidence. Superimposing twice-lifetime screening achieved 100% elimination in all these 3 groups [98].

Akin to the WHO, the Canadian Partnership Against Cancer [99] developed an action plan for a 90-90-90 target by 2030 to eliminate CC by 2040 projecting itself as the first nation to do so. Strategies include scaling up HPV vaccination, primary HPV screening and follow-up of abnormal results. In Australia, a modelling study revealed that courtesy of high coverage of vaccination and screening, the age-standardised annual incidence is <6/100,000 by 2020 and <4/100,000 by 2028. Regarding mortality, the trend indicates an age-standardised annual mortality rate of 1/100,000 [100]. The US-based Cancer Intervention and Surveillance Modelling Network predicted that by 2038–2046, a threshold CC incidence of < 4/100,000 is feasible. Elimination could be pre poned to 2028–2033 provided there is higher screening coverage [101].

### Cost effectiveness

When compared to visual inspection and cytology, HPV DNA cervical sampling is the most cost-efficient choice (Table 4). Not only is it the most affordable, but it also provides the greatest average reduction in cancer risk over a lifetime when compared to other tests. This can be illustrated from data collected in three countries – India, Nicaragua and Uganda. Indian analysis found that HPV Testing done thrice in a lifetime (at 30, 35 and 40 years) had an ICER of I$ 1600 per year of life saved (YLS) with the highest life expectancy gains and a reduction of 48.7% in lifetime risk of cancer. Similar statistics were observed in Nicaragua (I$1200 per YLS) and Uganda (I$420 per YLS) [110]. A Colombian study discovered cytology done at 3-year intervals to be the most expensive modality and HPV DNA testing repeated 5 years to be the cheapest along with an expected reduction in mortality from 69% to 81% [111]. An El Salvadorian study came to a similar conclusion that HPV Testing followed by cryotherapy was the most effective (reduction in cancer risk by 58.5%) and most cost-effective (US$490 per YLS) modality for CC screening [112]. A study conducted in Thailand assessed the cost effectiveness of using the detection of HPV p16/18 as the primary screening test as opposed to conventional cytology and found it to be a more sensitive (82.65% versus 55.85%, respectively) test while maintaining the specificity (96.47 versus 95.48%, respectively) and reduced false positives. All these factors make it a more cost-effective and well-rounded option with an ICER of $1395 per QALY gained [113].

The above evidence talks about the cost effectiveness of HPV testing by illustrating values of ICER per YLS and QALY while the following shines a light on the amount per detected lesion. According to the FOCAL trial conducted in Canada, HPV testing followed by reflexive cytology identified more lesions and was associated with a lower cost per detected lesion ($7551) as compared to the control group that received just cytology ($8325) [114]. Another study conducted in Thailand compared the ICER in terms of cost per detected case of CIN2 and found HPV testing to dominate over cytology. The cost per detected case was found to be $1219.9 [115]. A modelling study in the Netherlands concluded that the total costs fell by 21% and the cost per QALY gained was €12,225 (46% less) for HPV testing as compared to €22,678 for the cytology program [116]. Another HIC model-based study conducted by the NHS estimated annual savings of £13 million on average if a switch was made to HPV testing [117].

Even in settings where an established cytology-based screening program already exists, switching over to a hybrid strategy (cytology till age 29 and HPV Testing thereafter) will save funds with the money spent to obtain 1 year with the quality of life being under the willingness to pay threshold (negative ICER of US$ 37.87) [118].

### Coverage and effectiveness

The average percentage of 55 LMICs for women screened for CC was 44%. Much improvement needs to happen to reach the WHO 2030 goal of 70% screening coverage. Among the LMICs, Latin America and the Caribbean nations had the highest numbers (country-level median of 84.6% – IQR, 65.7%–91.1%; range, 11.7%–97.4%) while Sub-Saharan Africa lags severely behind with the global lowest screening coverage (country-level median, 16.9%; IQR, 3.7%–31.0%; range, 0.9%–50.8%) [119].

Some outliers from the LMIC group have shown an increasing trend in screening coverage despite many challenges. Notably, countries like Taiwan, Portugal, Mexico, Argentina and El Salvador show promising results. Africa, Botswana and Costa Rica have shown good progress. All these outliers have a well-executed population-based national screening program with HPV testing at the helm of it as the primary screening modality. The lifetime screening percentages for Botswana and El Salvador are 50% and 95%, respectively. Costa Rica has shown commendable growth in recent years with 77% of women having undergone screening in the past 5 years [6]. The success of the Latin American nations can be attributed to the presence of national CC screening programs that provide free-of-cost services to women at the local level [120]. Taiwan after implementing a national program has seen a reduction in invasive cancer by 47.8% during 1995–2006 [119]. Hence, while the developed nations predictably show an improving graph due to their higher expenditure, bridging the gap in LMICs is not an impossible task.

Regarding coverage for PLHIV, WHO guidelines provide special considerations. Testing commences from the age of 25 with a primary HPV DNA test. If positive, then a secondary test of either partial genotyping, colposcopy, VIA, or cytology is performed to triage women. This screen, triage and treat approach is to be repeated every 3–5 years till the age of 50 [74]. The factors that are shown to increase participation in screening programs are formal education, and knowledge and awareness of CC, its risk factors and severity. Women who had the aforementioned factors were around 3.5 times more likely to be screened [121]. Another fix could be the implementation of self-sampling for PLHIV as many are unable to access screening clinics due to HIV-associated stigma. Studies find it has good acceptability. Over 90% of PLHIV included in a study said self-sampling was comfortable and were willing to try it again. However, many also prefer clinician-collected samples as they trust trained professionals over themselves for sample collection [120–122].

The possible effect of vaccination on screening is noteworthy. According to Wilyman [123], HPV vaccination may decrease the rate of abnormal Pap tests and ultimately weaken the positive predictive value of cytology. Hence, once HPV vaccination becomes a routine practice, the HPV test may be considered as a primary screening procedure and cytology for women thereafter who test HPV positive. Other considerations may include delaying the initial screening to 25 years of age and reducing the frequency of screening [123].

A substantial challenge to CC screening in recent times has been the COVID-19 pandemic. Screening rates plummeted by 94% post movement restriction orders. After lifting restrictions, the screening numbers were 35% less than pre-pandemic trends. A comparative model-based analysis done to assess the future impact of COVID-19 disruptions showed that women screened with cytology alone would show the greatest increase in cancer incidence [124]. It also revealed that delaying screening in previously HPV-negative women would not have a significant impact. A risk-based approach for prioritising patients (versus an algorithm-based strategy), with an HPV-based screening program with special emphasis on self-collected HPV testing and integration of telehealth is recommended [125]. Having mentioned the aforesaid, in the setting of a fear of entering public spaces, HPV SS testing kits delivered at the doorstep are predicted to increase the population uptake of screening [89].

### People living with HIV

CC screening guidelines for PLHIV differ slightly from the general population due to their increased risk of developing CC [126].

The American College of Obstetricians and Gynecologists (ACOG) recommends initiating screening earlier for HIV-positive women, starting at age 21 [127]. This recommendation aligns with guidelines from other organisations like the CDC [128].

Screening frequency may be increased compared to the general population. ACOG suggests Pap smears every 6 months for the first 2 years followed by yearly Pap smears or co-testing (Pap smear and HPV test) every 3 years thereafter, depending on individual risk factors [127]. The CDC also suggests similar increased screening frequency for high-risk individuals, including those with HIV [128].

HPV testing may play a more prominent role in screening for HIV-positive individuals. Co-testing is being increasingly recommended as it can improve the detection of pre-cancerous lesions even in the presence of abnormal Pap smears that might be misleading due to immune system issues in HIV [127, 129, 130].

Early detection and intervention are crucial for preventing CC in this high-risk population. The adapted screening guidelines aim to balance the need for increased vigilance with minimizing unnecessary procedures while considering the potential for inconclusive Pap smears due to immune system factors.

## Conclusion

The dramatic disparity in incidence rates between HICs and LMICs is primarily due to differential access to effective screening and preventive treatment; similar disparities also exist within countries. As HPV vaccinations are practically feasible and cost-effective, increasing acceptance and coverage should be a public health priority. While the context-specific barriers should be addressed, there is a clear need for regular monitoring and sustained funding within all HIC and LMIC settings to ensure progress is sustained. The efficacy of single-dose schedules has the potential to accelerate progress considerably in the coming years; they will massively enhance cost-effectiveness, simplify delivery schedules and enhance coverage. On another note, HPV testing for primary screening dominates other strategies (i.e., it is more effective and cost effective) and, therefore, if implemented properly with regular testing and stringent quality assurance checks, it could become a well-rounded and universal option with cost-effectiveness and optimum therapeutic value for LMICs and HICs alike. However, vaccines should not substitute screening and vice-versa – a combined approach is needed to combat this global problem. It is undoubtedly the need of the hour as international gaps are threatened to widen secondary to the lag in LMICs.

## Conflicts of interest

No conflict of interest.

## Funding

No funding.

## Author contributions

Conceptualisation and design – AG, KW, SS, BS, SM.

Data collection and assembly – IM, AK, LM, AG, SB1, SB2.

Data analysis and interpretation - IM, AK, LM, AG, KW, NSD, SS, BS, JST, SM.

Manuscript writing – IM, AK, LM, KW, NSD.

Final approval of manuscript – All authors.

## Figures and Tables

**Table 1.** This table demonstrates characteristics of currently licenced HPV prophylactic vaccinations [15–17, 25, 53].

Vaccine brand name	Year of licensure	HPV strains covered	Other diseases covered	Price/ dose
Gardasil, Silgard (Merck and Co)	2006	Quadrivalent: 6, 11, 16, 18	Anogenital cancers (vulval, vaginal, anal cancers), some head and neck cancers, laryngeal papillomas, genital warts	Approx €110
Cervarix (Glaxosmithkline)	2009	Bivalent: 16, 18	Anogenital cancers (vulval, vaginal, anal cancers), some head and neck cancers	Approx €90
Gardasil-9 (Merck and Co)	2014	Nonavalent: 6, 11, 16, 18, 31, 33, 45, 52, 58	Same as Gardasil but broader protection due to additional strains covered	Approx €200
Cecolin (Xiamen Innovac)	2020	Bivalent: 16, 18	Similar to other bivalent vaccines	Approx €40
Walvax recombinant HPV vaccine (Shanghai Zerun Biotech)	2022	Bivalent: 16, 18	Similar to other bivalent vaccines	Approx €40
Cervavac (Serum Institute of India and Dep of Biotech)	2022	Quadravalent: 6, 11, 16, 18	Likely comparable to the other quadrivalent vaccine	Approx €4

**Table 2.** This table summarises the pooled sensitivity and specificity of the various screening strategies discussed [66, 79, 93].

Testing strategy	Sensitivity	Specificity
HPV DNA testing	0.94 (95% CI 0.89–0.97)	0.88 (95% CI 0.84–0.92)
HPV SS	0.76 (95% CI 0.69–0.82) for CIN2+ 0.84 (95% CI 0.72–0.92) for CIN3+	0.86 (95% CI 0.83–0.89) for CIN2+ 0.87 (95% CI 0.84–0.90) for CIN3+
Co-testing	0.95 (95% CI 0.92–1) for CIN2+ 0.97 (95% CI 0.94–1) for CIN3+	0.95 (95% CI 0.94–0.96) for CIN2+ 0.93 (95% CI 0.92–0.95) for CIN3+
Cytology	0.70 (95% CI 0.57–0.80)	0.95 (95% CI 0.92–0.97)
VIA	0.69 (95% CI 0.54–0.81)	0.87 (95% CI 0.79–0.92

**Table 3.** This table summarises the testing protocol of population-based screening programs in select HICs and LMICs [102–109].

Sl No.	Country/ Region/ Organisation		Age range	Testing protocol
1.	United States of America	American College of Gynaecology	21–65	Cytology only, every 3 years, till age of 29. Followed by one of the following: I. HPV testing every 5 years II. PAP smear every 3 years III. HPV testing with PAP smear every 5 years
		United States Preventive Services Task Force	21–65	
		American Cancer Society	25–65	
2.	Europe		25 to 60–65	Cytology only, every 3–5 years, till age of 35 followed by HPV testing every 5 years
3.	United Kingdom		25–64	HPV testing, every 3 years, till age of 49 followed by testing every 5 years.
4.	Australia		25–74	HPV testing every 5 years.
5.	Asia	India	30–65	VIA every 5 years.
		Bhutan/Maldives/Sri Lanka	20–60	Cytology/VIA every 3–5 years.
		Thailand	20–60	HPV testing every 5 years.
6.	WHO Africa		25–65	Cytology/VIA every 3–5 years.

**Table 4.** This table demonstrates the cost effectiveness of HPV testing strategies over cytology [110–116].

Screening strategy	Testing interval	Country & year	Cost effectiveness when compared to cytology (ICER)
HPV DNA testing thrice in a lifetime	30, 35, 40 years 30, 35, 40 years 25, 35, 45 years	India (2015) Nicargua (2015) Uganda (2015)	I$ 1600 per YLS with 48.7% reduction in cancer risk I$ 1200 per YLS with 53.5% reduction in cancer risk I$ 420 per YLS with 50.9% reduction in cancer risk
HPV testing followed by cryotherapy	Testing every 5 years for ages between 30 and 65	El Salvador (2019)	US$490 per YLS with 58.5% reduction in cancer risk
HPV p16/18 detection	Testing every 5 years for ages between 30 and 65	Thailand (2019)	$1395 per QALY gained
HPV DNA testing	Testing every 5 years for ages between 30 and 60	Netherlands (2021)	€12225 per QALY Gained
HPV DNA testing	Testing every 5 years after 35 years	Thailand (2017)	$1219.9 per detected CIN2+ lesion
HPV testing followed by reflexive cytology	Testing every 4 years	Canada (2021)	$7551 per detected lesion

## References

[1] Sung H, Ferlay J, Siegel RL (2021). Global cancer statistics 2020: GLOBOCAN estimates of incidence and mortality worldwide for 36 cancers in 185 countries. CA Cancer J Clin.

[2] Bosch FX, Burchell AN, Schiffman M (2008). Epidemiology and natural history of human papillomavirus infections and type-specific implications in cervical neoplasia. Vaccine.

[3] IARC Working Group on the Evaluation of Carcinogenic Risks to Humans (2007). Human papillomaviruses. IARC Monogr Eval Carcinog Risks Hum.

[4] Cohen PA, Jhingran A, Oaknin A (2019). Cervical cancer. Lancet.

[5] Ferlay J, Colombet M, Soerjomataram I (2021). Cancer statistics for the year 2020: an overview. Int J Cancer.

[6] World Health Organization (2020). Global Strategy to Accelerate the Elimination of Cervical Cancer as a Public Health Problem.

[7] Arbyn M, Weiderpass E, Bruni L (2020). Estimates of incidence and mortality of cervical cancer in 2018: a worldwide analysis. Lancet Glob Health.

[8] Gultekin M, Ramirez PT, Broutet N (2020). World Health Organization call for action to eliminate cervical cancer globally. Int J Gynecol Cancer.

[9] Bruni L, Saura-Lázaro A, Montoliu A (2021). HPV vaccination introduction worldwide and WHO and UNICEF estimates of national HPV immunization coverage 2010–2019. Prev Med.

[10] World Health Organization (2014). Comprehensive Cervical Cancer Control: a Guide to Essential Practice.

[11] Drolet M, Pérez N (2019). Population-level impact and herd effects following the introduction of human papillomavirus vaccination programmes: updated systematic review and meta-analysis. Lancet.

[12] de Martel C, Plummer M, Vignat J (2017). Worldwide burden of cancer attributable to HPV by site, country and HPV type. Int J Cancer.

[13] Cervical Cancer.

[14] Senkomago V, Henley SJ, Thomas CC (2019). Human papillomavirus-attributable cancers – United States, 2012–2016. MMWR Morb Mortal Wkly Rep.

[15] GlaxoSmithKline (2023). FDA Approves Cervarix, GlaxoSmithKline’s Cervical Cancer Vaccine.

[16] Centres for Disease Control and Prevention (2024). Human Papillomavirus (HPV) Vaccine.

[17] Zou Z, Fairley CK, Ong JJ (2020). Domestic HPV vaccine price and economic returns for cervical cancer prevention in China: a cost-effectiveness analysis. Lancet Glob Health.

[18] Dehlendorff C, Sparén P, Baldur-Felskov B (2018). Effectiveness of varying number of doses and timing between doses of quadrivalent HPV vaccine against severe cervical lesions. Vaccine.

[19] Kim JJ, Simms KT, Killen J (2021). Human papillomavirus vaccination for adults aged 30 to 45 years in the United States: a cost-effectiveness analysis. PLoS Med.

[20] Newman PA, Logie CH, Doukas N (2013). HPV vaccine acceptability among men: a systematic review and meta-analysis. Sex Transm Infect.

[21] World Health Organization (2016). Guide to Introducing HPV Vaccine Into National Immunization Programmes.

[22] Human Papillomavirus (HPV) Vaccination Coverage.

[23] World Health Organization (2022). Highlights from the Meeting of the Strategic Advisory Group of Experts (SAGE) on Immunization.

[24] Arbyn M, Xu L, Simoens C (2018). Prophylactic vaccination against human papillomaviruses to prevent cervical cancer and its precursors. Cochrane Database Syst Rev.

[25] Bergman H, Buckley BS, Villanueva G (2019). Comparison of different human papillomavirus (HPV) vaccine types and dose schedules for prevention of HPV-related disease in females and males. Cochrane Database Syst Rev.

[26] Yang DY, Bracken K (2016). Update on the new 9-valent vaccine for human papillomavirus prevention. Can Fam Physician.

[27] Barnabas RV, Brown ER, Onono MA (2022). Efficacy of single-dose HPV vaccination among young African women. NEJM Evid.

[28] Watson-Jones D, Changalucha J, Whitworth H (2022). Immunogenicity and safety of one-dose human papillomavirus vaccine compared with two or three doses in Tanzanian girls (DoRIS): an open-label, randomised, non-inferiority trial. Lancet Glob Health.

[29] (2017). Meeting of the global advisory committee on vaccine safety, 7–8 June 2017. Wkly Epidemiol Rec.

[30] UNICEF (2019). Human Papillomavirus Vaccine: Supply and Demand Update.

[31] Falcaro M, Castañon A, Ndlela B (2021). The effects of the national HPV vaccination programme in England, UK, on cervical cancer and grade 3 cervical intraepithelial neoplasia incidence: a register-based observational study. Lancet.

[32] Simms KT, Hanley SJB, Smith MA (2020). Impact of HPV vaccine hesitancy on cervical cancer in Japan: a modelling study. Lancet Public Health.

[33] Ministry of Health, Labour and Welfare of Japan (2023). HPV Vaccine Adverse Event Reports in the National Committee on Vaccine Safety.

[34] Gallagher KE, Howard N, Kabakama S (2017). Lessons learnt from human papillomavirus (HPV) vaccination in 45 low- and middle-income countries. PLoS One.

[35] Spayne J, Hesketh T (2021). Estimate of global human papillomavirus vaccination coverage: analysis of country-level indicators. BMJ Open.

[36] Dorji T, Nopsopon T, Tamang ST (2021). Human papillomavirus vaccination uptake in low-and middle-income countries: a meta-analysis. EClinicalMedicine.

[37] World Cancer Research Fund International (2024). Cervical Cancer Statistics.

[38] Wilson R (2021). HPV vaccine acceptance in West Africa: a systematic literature review. Vaccine.

[39] Wijayanti KE, Schütze H, MacPhail C (2021). Parents' knowledge, beliefs, acceptance and uptake of the HPV vaccine in members of The Association of Southeast Asian Nations (ASEAN): a systematic review of quantitative and qualitative studies. Vaccine.

[40] Silva TM, Nogueira de Sá AC, Beinner MA (2022). Impact of the COVID-19 pandemic on human papillomavirus vaccination in Brazil. Int J Public Health.

[41] Rai Y, Webster H, Tessier E (2020). Human papillomavirus (HPV) vaccination coverage in adolescent females and males in England: academic year 2019 to 2020. Health Prot Rep.

[42] Daniels V, Saxena K, Roberts C (2021). Impact of reduced human papillomavirus vaccination coverage rates due to covid-19 in the United States: a model based analysis. Vaccine.

[43] WHO Immunization Data Portal.

[44] Lavie M, Lavie I, Laskov I (2023). Impact of Covid-19 pandemic on human papillomavirus vaccine uptake in Israel. J Low Genit Tract Dis.

[45] Smith DL, Perkins RB (2022). Low rates of HPV vaccination and cervical cancer screening: challenges and opportunities in the context of the COVID-19 pandemic. Prev Med.

[46] Considerations for Integrating COVID-19 Vaccination with Other Childhood and Adolescent Immunizations: Interim Guidance.

[47] Rosettie KL, Joffe JN, Sparks GW (2021). Cost-effectiveness of HPV vaccination in 195 countries: a meta-regression analysis. PLoS One.

[48] Tran PT, Riaz M, Chen Z (2022). An umbrella review of the cost effectiveness of human papillomavirus vaccines. Clin Drug Investig.

[49] Datta S, Pink J, Medley GF (2019). Assessing the cost-effectiveness of HPV vaccination strategies for adolescent girls and boys in the UK. BMC Infect Dis.

[50] Prem K, Choi YH (2021). Global impact and cost-effectiveness of one-dose versus two-dose human papillomavirus vaccination schedules: a comparative modelling analysis. MedRxiv.

[51] Jiang Y, Ni W, Wu J (2019). Cost-effectiveness and value-based prices of the 9-valent human papillomavirus vaccine for the prevention of cervical cancer in China: an economic modelling analysis. BMJ Open.

[52] Van Minh H, My NTT, Jit M (2017). Cervical cancer treatment costs and cost-effectiveness analysis of human papillomavirus vaccination in Vietnam: a PRIME modeling study. BMC Health Serv Res.

[53] The Lancet Oncology (2022). HPV vaccination in south Asia: new progress, old challenges. Lancet Oncol.

[54] Burki TK (2023). India rolls out HPV vaccination. Lancet Oncol.

[55] World Health Organization (2020). Human Papillomavirus (HPV) Vaccines: WHO Position Paper.

[56] Centers for Disease Control and Prevention (2023). HPV Vaccination for People with HIV.

[57] BHIVA guidelines on the use of vaccines in HIV-positive adults 2015. https://www.bhiva.org/vaccination-guidelines.

[58] Losada C, Samaha H, Scherer EM (2023). Efficacy and durability of immune response after receipt of HPV vaccines in people living with HIV. Vaccines.

[59] Staadegaard L, Rönn MM, Soni N (2022). Immunogenicity, safety, and efficacy of the HPV vaccines among people living with HIV: a systematic review and meta-analysis. eClinicalMedicine.

[60] Zizza A, Banchelli F, Guido M (2021). Efficacy and safety of human papillomavirus vaccination in HIV-infected patients: a systematic review and meta-analysis. Sci Rep.

[61] (2012). Human papillomavirus. https://www.ecdc.europa.eu/en/human-papillomavirus.

[62] Johnson N (2020). Comprehensive sexual health assessments for adolescents. Paediatr Child Health.

[63] Shieh Y, Eklund M, Sawaya GF (2016). Population-based screening for cancer: hope and hype. Nat Rev Clin Oncol.

[64] Safaeian M, Solomon D, Castle PE (2007). Cervical cancer prevention – cervical screening: science in evolution. Obstet Gynecol Clin North Am.

[65] Mayrand MH, Duarte-Franco E, Rodrigues I (2007). Human papillomavirus DNA versus Papanicolaou screening tests for cervical cancer. N Engl J Med.

[66] Mustafa RA, Santesso N, Khatib R (2016). Systematic reviews and meta-analyses of the accuracy of HPV tests, visual inspection with acetic acid, cytology, and colposcopy. Int J Gynaecol Obstet.

[67] Catarino R, Petignat P, Dongui G (2015). Cervical cancer screening in developing countries at a crossroad: emerging technologies and policy choices. World J Clin Oncol.

[68] Sankaranarayanan R, Budukh AM, Rajkumar R (2001). Effective screening programmes for cervical cancer in low- and middle-income developing countries. Bull World Health Organ.

[69] Bedell SL, Goldstein LS, Goldstein AR (2020). Cervical cancer screening: past, present, and future. Sex Med Rev.

[70] Srivastava AN, Misra JS, Srivastava S (2018). Cervical cancer screening in rural India: status & current concepts. Indian J Med Res.

[71] Silkensen SL, Schiffman M, Sahasrabuddhe V (2018). Is it time to move beyond visual inspection with acetic acid for cervical cancer screening?. Glob Health Sci Pract.

[72] Vedantham H, Silver MI, Kalpana B (2010). Determinants of VIA (Visual inspection of the cervix after acetic acid application) positivity in cervical cancer screening of women in a Peri-urban area in Andhra Pradesh, India. Cancer Epidemiol Biomarkers Prev.

[73] Bosch FX, Lorincz A, Muñoz N (2002). The causal relation between human papillomavirus and cervical cancer. J Clin Pathol.

[74] World Health Organization (2021). WHO Guideline for Screening and Treatment of Cervical Pre-Cancer Lesions for Cervical Cancer Prevention.

[75] Hillyar CR, Kanabar SS, Pufal KR (2022). A systematic review and meta-analysis of the diagnostic effectiveness of human papillomavirus methylation biomarkers for detection of cervical cancer. Epigenomics.

[76] Ronco G, Dillner J, Elfström KM (2014). Efficacy of HPV-based screening for prevention of invasive cervical cancer: follow-up of four European randomised controlled trials. Lancet.

[77] Sankaranarayanan R, Nene BM, Shastri SS (2009). HPV screening for cervical cancer in rural India. N Engl J Med.

[78] Wright TC, Stoler MH, Behrens CM (2015). Primary cervical cancer screening with human papillomavirus: end of study results from the ATHENA study using HPV as the first-line screening test. Gynecol Oncol.

[79] Arbyn M, Ronco G, Anttila A (2012). Evidence regarding human papillomavirus testing in secondary prevention of cervical cancer. Vaccine.

[80] Schiffman M, Kinney WK, Cheung LC (2018). Relative performance of HPV and cytology components of cotesting in cervical screening. J Natl Cancer Inst.

[81] Liang LA, Einzmann T, Franzen A (2021). Cervical cancer screening: comparison of conventional Pap Smear Test, liquid-based cytology, and human papillomavirus testing as stand-alone or cotesting strategies. Cancer Epidemiol Biomarkers Prev.

[82] Gage JC, Schiffman M, Katki HA (2014). Reassurance against future risk of precancer and cancer conferred by a negative human papillomavirus test. J Natl Cancer Inst.

[83] Ratnam S, Coutlee F, Fontaine D (2011). Aptima HPV E6/E7 mRNA test is as sensitive as hybrid capture 2 assay but more specific at detecting cervical precancer and cancer. J Clin Microbiol.

[84] Arbyn M, Simon M, Sanjosé S (2022). Accuracy and effectiveness of HPV mRNA testing in cervical cancer screening: a systematic review and meta-analysis. Lancet Oncol.

[85] Sørbye SW, Fismen S, Gutteberg TJ (2016). Primary cervical cancer screening with an HPV mRNA test: a prospective cohort study. BMJ Open.

[86] Maggino T, Sciarrone R, Murer B (2016). Screening women for cervical cancer carcinoma with a HPV mRNA test: first results from the Venice pilot program. Br J Cancer.

[87] Giorgi Rossi P, Ronco G, Mancuso P (2022). Performance of HPV E6/E7 mRNA assay as primary screening test: results from the NTCC2 trial. Int J Cancer.

[88] Zappacosta R, Gatta DM, Marinucci P (2015). Role of E6/E7 mRNA test in the diagnostic algorithm of HPV-positive patients showing ASCUS and LSIL: clinical and economic implications in a publicly financed healthcare system. Expert Rev Mol Diagn.

[89] Yeh PT, Kennedy CE, Vuyst H (2019). Self-sampling for human papillomavirus (HPV) testing: a systematic review and meta-analysis. BMJ Glob Health.

[90] Regional Committee for Europe, 72nd session (2022). Seventy-Second Regional Committee for Europe: Tel Aviv, 12–14 September 2022: Roadmap to Accelerate the Elimination of Cervical Cancer as a Public Health Problem in the WHO European Region 2022–2030.

[91] Bais AG, Kemenade FJ, Berkhof J (2007). Human papillomavirus testing on self-sampled cervicovaginal brushes: an effective alternative to protect nonresponders in cervical screening programs. Int J Cancer.

[92] Wong EL, Cheung AW, Wong AY (2020). Acceptability and feasibility of HPV self-sampling as an alternative primary cervical cancer screening in under-screened population groups: a cross-sectional study. Int J Environ Res Public Health.

[93] Arbyn M, Verdoodt F, Snijders PJ (2014). Accuracy of human papillomavirus testing on self-collected versus clinician-collected samples: a meta-analysis. Lancet Oncol.

[94] Gupta S, Palmer C, Bik EM (2018). Self-sampling for human papillomavirus testing: increased cervical cancer screening participation and incorporation in International Screening Programs. Front Public Health.

[95] Lazcano-Ponce E, Lorincz AT, Cruz-Valdez A (2011). Self-collection of vaginal specimens for human papillomavirus testing in cervical cancer prevention (MARCH): a community-based randomised controlled trial. Lancet.

[96] Wang W, Arcà E, Sinha A (2022). Cervical cancer screening guidelines and screening practices in 11 countries: a systematic literature review. Prev Med Rep.

[97] Canfell K, Kim JJ, Brisson M (2020). Mortality impact of achieving WHO cervical cancer elimination targets: a comparative modelling analysis in 78 low-income and lower-middle-income countries. Lancet.

[98] Brisson M, Kim JJ, Canfell K (2020). Impact of HPV vaccination and cervical screening on cervical cancer elimination: a comparative modelling analysis in 78 low-income and lower-middle-income countries. Lancet.

[99] Canadian Partnership Against Cancer (2024). Action Plan for the Elimination of Cervical Cancer in Canada, 2020–2030.

[100] Hall MT, Simms KT, Lew JB (2019). The projected timeframe until cervical cancer elimination in Australia: a modelling study. Lancet Public Health.

[101] Burger EA, Smith MA, Killen J (2020). Projected time to elimination of cervical cancer in the USA: a comparative modelling study. Lancet Public Health.

[102] Curry SJ, Krist AH, US Preventive Services Task Force (2018). Screening for cervical cancer: US preventive services task force recommendation statement. JAMA.

[103] American College of Obstetricians and Gynecologists (2021). Updated Cervical Cancer Screening Guidelines. Practice Advisory.

[104] American Cancer Society (2021). The American Cancer Society Guidelines for the Prevention and Early Detection of Cervical Cancer.

[105] von Karsa L, Arbyn M, De Vuyst H (2015). European guidelines for quality assurance in cervical cancer screening. Summary of the supplements on HPV screening and vaccination. Papillomavirus Res.

[106] Australian Institute of Health and Welfare (2021). Cancer in Australia 2021. Cancer Series no. 133. Cat. no. CAN 144.

[107] Public Health England (2021). NHS Cervical Screening (CSP) Programme: Detailed Information.

[108] IARC Working Group on the Evaluation of Cancer-Preventive Interventions (2022). IARC Handbooks of Cancer Prevention, Volume 18: Cervical Cancer Screening.

[109] Ministry of Health and Family Welfare, Government of India (2016). Operational Framework – Management of Common Cancers.

[110] Campos NG, Tsu V, Jeronimo J (2015). When and how often to screen for cervical cancer in three low- and middle-income countries: a cost-effectiveness analysis. Papillomavirus Res.

[111] Andrés-Gamboa O, Chicaíza L, García-Molina M (2008). Cost-effectiveness of conventional cytology and HPV DNA testing for cervical cancer screening in Colombia. Salud Publica Mex.

[112] Campos NG, Maza M, Alfaro K (2019). The cost-effectiveness of implementing HPV testing for cervical cancer screening in El Salvador. Int J Gynaecol Obstet.

[113] Termrungruanglert W, Khemapech N, Tantitamit T (2019). Cost effectiveness analysis of HPV primary screening and dual stain cytology triage compared with cervical cytology. J Gynecol Oncol.

[114] Cromwell I, Smith LW, Hoek K (2021). Cost-effectiveness analysis of primary human papillomavirus testing in cervical cancer screening: results from the HPV FOCAL trial. Cancer Med.

[115] Termrungruanglert W, Khemapech N, Tantitamit T (2017). Cost-effectiveness analysis study of HPV testing as a primary cervical cancer screening in Thailand. Gynecol Oncol Rep.

[116] Jansen E, Naber SK, Aitken CA (2021). Cost-effectiveness of HPV-based cervical screening based on first year results in the Netherlands: a modelling study. BJOG.

[117] Bains I, Choi YH, Soldan K (2019). Clinical impact and cost-effectiveness of primary cytology versus human papillomavirus testing for cervical cancer screening in England. Int J Gynecol Cancer.

[118] Vale DB, Silva MT, Discacciati MG (2021). Is the HPV-test more cost-effective than cytology in cervical cancer screening? An economic analysis from a middle-income country. PLoS One.

[119] Lemp JM, De Neve JW, Bussmann H (2020). Lifetime prevalence of cervical cancer screening in 55 low- and middle-income countries. JAMA.

[120] National Cancer Institute: Surveillance, Epidemiology and End Results Program (2024). Cancer Stat Facts: Cervical Cancer.

[121] Dessalegn Mekonnen B (2020). Cervical cancer screening uptake and associated factors among HIV-positive women in Ethiopia: a systematic review and meta-analysis. Adv Prev Med.

[122] Asare M, Abah E, Obiri-Yeboah D (2022). HPV self-sampling for cervical cancer screening among women living with HIV in low- and middle-income countries: what do we know and what can be done?. Healthcare (Basel).

[123] Wilyman J (2013). HPV vaccination programs have not been shown to be cost-effective in countries with comprehensive Pap screening and surgery. Infect Agent Cancer.

[124] Burger EA, Sy S, Kim JJ (2020). Impact of COVID-19-related care disruptions on cervical cancer screening in the United States, annual society of medical decision making meeting (Virtual).

[125] Wentzensen N, Clarke MA, Perkins RB (2021). Impact of COVID-19 on cervical cancer screening: challenges and opportunities to improving resilience and reduce disparities. Prev Med.

[126] Lacey CJN (2019). HPV vaccination in HIV infection. Papillomavirus Res.

[127] Updated cervical cancer screening guidelines. https://www.acog.org/clinical/clinical-guidance/practice-advisory/articles/2021/04/updated-cervical-cancer-screening-guidelines.

[128] HPV Vaccination Recommendations.

[129] Cervical cancer-health professional version. https://www.cancer.gov/types/cervical/hp.

[130] (2023). Cervical Cancer.

